# Predisposing conditions in patients with small intestinal adenocarcinomas in the Netherlands: A 20‐year nationwide cohort study

**DOI:** 10.1002/ijc.35354

**Published:** 2025-02-05

**Authors:** Jasmijn D. G. Linssen, Pascale J. M. Schafrat, Tim R. de Back, Felice N. van Erning, Monique E. van Leerdam, Evelien Dekker, Louis Vermeulen, Ignace H. J. T. de Hingh, Dirkje W. Sommeijer

**Affiliations:** ^1^ Cancer Center Amsterdam, Laboratory for Experimental Oncology and Radiobiology Center for Experimental and Molecular Medicine Amsterdam The Netherlands; ^2^ Amsterdam Gastroenterology Endocrinology Metabolism, Laboratory for Experimental Oncology and Radiobiology Center for Experimental and Molecular Medicine Amsterdam The Netherlands; ^3^ Oncode Institute Utrecht The Netherlands; ^4^ Department of Gastroenterology and Hepatology Amsterdam UMC, Location University of Amsterdam Amsterdam The Netherlands; ^5^ Department of Medical Oncology Amsterdam UMC, Location University of Amsterdam Amsterdam The Netherlands; ^6^ Department of Surgery Catharina Hospital Eindhoven The Netherlands; ^7^ Department of Research and Development Netherlands Comprehensive Cancer Organization (IKNL) Utrecht The Netherlands; ^8^ Department of Gastroenterology and Hepatology Leiden University Medical Center Leiden The Netherlands; ^9^ Department of Gastrointestinal Oncology Netherlands Cancer Institute Amsterdam The Netherlands; ^10^ Department of Epidemiology, GROW‐School for Oncology and Developmental Biology Maastricht University Maastricht The Netherlands; ^11^ Department of Internal Medicine Flevohospital Almere The Netherlands

**Keywords:** incidence, predisposing conditions, prognosis, small intestinal adenocarcinoma

## Abstract

Small intestinal adenocarcinomas (SIAs) are associated with predisposing conditions, including inflammatory bowel disease (IBD) and celiac disease, but also genetic syndromes such as Lynch syndrome (LS) and familial adenomatous polyposis (FAP). This nationwide cohort study investigated the incidence of genetic and non‐genetic predisposing conditions in SIA and their influence on tumor characteristics and clinical features. Data were obtained from the Netherlands Cancer Registry. The incidence, characteristics, and clinical features per predisposing condition were analyzed in 2697 SIA patients diagnosed from 1999 through 2019. Of all SIA patients, 5.6% were known to have a genetic predisposing syndrome, of whom 4.0% had LS and 1.6% had a polyposis syndrome. In addition, 6.8% of SIA patients had a non‐genetic predisposing condition: 3.9% IBD and 2.9% celiac disease. SIAs of patients with such predisposing syndromes or conditions were diagnosed at a younger age and earlier stage and affected the duodenum less often as compared to sporadic SIA patients. Both genetic and non‐genetic predisposing conditions were associated with significantly better overall survival (OS) compared to sporadic SIA: sporadic SIA (median OS: 13.0 months, 95% CI: 11.8–14.2), LS (213.1 months, 99.3–NA), polyposis syndromes (61.3 months, 19.7–NA), IBD (29.5 months, 20.3–69.8), and celiac disease (50.4 months, 24.6–124.7). This nationwide cohort study shows significant differences between SIA with and without predisposing conditions and highlights the need for research on underlying molecular mechanisms to improve outcomes of SIA patients.

## Graphical abstract

What's new?

Although small intestinal adenocarcinoma (SIA) shares certain similarities with colorectal cancer, its etiology and predisposing conditions presumably are distinct. Due to the rarity of SIA, however, detailed information on predisposing factors is lacking. Here, using data from the Netherlands Cancer Registry, the authors analyzed predisposing conditions and their influence on tumor characteristics in nearly 2700 SIA patients. Analyses show that 5.6 percent and 6.8 percent of patients had either a genetic predisposing syndrome or non‐genetic predisposing condition, respectively. The remainder of cases were sporadic and, compared to SIA with predisposing factors, were associated with worse overall survival.

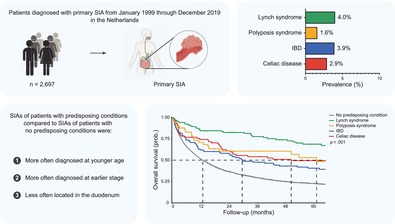

## 1 INTRODUCTION

Small intestinal cancers are rare and represent less than 5% of all gastrointestinal tumors.^1^, ^2^, ^3^, ^4^ The most common histological subtype (40%) is small intestinal adenocarcinoma (SIA).^2^, ^5^, ^6^, ^7^, ^8^ In Europe, approximately 3600 new patients are diagnosed with SIA annually.^9^ Over the past decades, the incidence of SIA is rising in Europe and the United States, mainly due to a growing number of patients with duodenal adenocarcinomas.^2^, ^3^, ^7^, ^10^, ^11^ Nevertheless, SIA has an unfavorable prognosis with 5‐year survival rates of 57%–79% for stage I and 3%–19% for stage IV disease, without improvement over the past decades.^2^, ^6^, ^11^, ^12^, ^13^

Despite similarities in tissue of origin with colorectal cancer (CRC), the etiology and predisposing conditions of SIA are postulated to be distinct.^14^ Known genetic predispositions associated with SIA include Lynch syndrome (LS), familial adenomatous polyposis (FAP), and Peutz–Jeghers syndrome (PJS). Other underlying diseases associated with SIA are inflammatory bowel disease (IBD) and celiac disease.^14^, ^15^ The prevalence of these conditions is suggested to be higher in SIA compared to CRC.^16^, ^17^, ^18^ Probably due to the rarity of SIA, observed results display large variations among studies. A French cohort (*n* = 346) reported a prevalence of a predisposing condition in 19.7% of SIA patients, while a Japanese study (*n* = 205) observed a prevalence of 1.5%.^8^, ^15^ Currently, large nationwide cohort studies are lacking, and therefore precise incidence rates of predisposing conditions and their correlation with tumor type are limited.

This critical lack of data, coupled with the increasing incidence of SIA and its poor survival, makes it pivotal to acquire a deeper understanding of predisposing conditions and their relation to tumor characteristics. The aim of this retrospective population‐based study is to gain more insight into the incidence of predisposing conditions in SIA and their impact on disease course and other clinical features.

## 2 METHODS

### 2.1 Data source and study population

This study represents all adult patients diagnosed with a primary SIA from January 1999 through December 2019 in the Netherlands. Clinical data were obtained from the Netherlands Cancer Registry (NCR), complemented with pathological data retrieved from the Dutch pathology register PALGA.^19^ In this study, we included only patients diagnosed with a primary adenocarcinoma of the small intestine, according to the International Classification of Disease for Oncology, code C17.^20^ Patients with small intestinal tumors of non‐adenocarcinoma histology or unknown histology were excluded from the cohort. For stage IV SIA patients, the NCR only records synchronous metastases. Systemic therapy data has been tracked since 2015. Patient vital status was obtained from the Dutch municipal Personal Records Database (BRP). Further detailed information on data collection and study population was recently published.^11^ Additionally, to obtain data on pathogenic germline variants in mismatch repair (MMR) genes (*MLH1*, *MSH2*, *MSH6*, and *PMS2*) for individuals with LS, pathology reports were linked with the Netherlands Foundation for Detection of Hereditary Tumours, StOET. If a patient was not registered within the Netherlands Foundation for Detection of Hereditary Tumours, PALGA records were reviewed. As patient data were anonymized, patient consent was not required.

Pathological records from PALGA were reviewed to collect clinical or genetically proven data on predisposing conditions, including Crohn's disease (CD), ulcerative colitis (UC), IBD of unknown origin (IBD‐U), celiac disease, LS, FAP, MUTYH‐associated polyposis (MAP), attenuated FAP, polyposis not otherwise specified, PJS, and juvenile polyposis. In case of uncertainty about the diagnosis of a predisposing condition, pathology reports were independently re‐examined by two researchers (JDGL, PJMS) to reach consensus. If necessary, an experienced oncologist (DWS) or gastroenterologist (ED) was consulted. Subsequently, predisposing conditions were subcategorized into four groups: (1) IBD, compromising CD, UC, and IBD‐U; (2) celiac disease; (3) LS; and (4) polyposis syndromes, containing FAP, MAP, PJS, attenuated FAP, juvenile polyposis, and polyposis not otherwise specified.

### 2.2 Statistical analyses

Patient and tumor characteristics were studied per group and per predisposing condition. Patients with a double diagnosis of a predisposing condition were excluded from any analysis.

Standard descriptive statistics were used to assess patient characteristics. The *χ* ^2^ test or the Fisher's exact test was used to compare categorical variables. Continuous variables were analyzed using independent sample *T*‐tests. Overall survival (OS) was defined as the time from diagnosis until death from any cause. Kaplan–Meier survival curves were conducted for the total cohort according to predisposing condition group and separately for each predisposing condition group according to disease stage. Differences were evaluated by log‐rank statistics. Univariable and multivariable Cox regression analyses were utilized for the total cohort and per predisposing condition group. For multivariable analyses, the assumption of proportional hazards per variable was met, and only variables that reached the significant level in the univariable analysis were included. Variables with missing cases were excluded in multivariable models. All tests were two‐sided, and a *p*‐value <.05 was considered statistically significant. Statistical analyses were performed using RStudio, version 4.2.1, and SPSS statistical software, version 28.0.1.1.

## 3 RESULTS

### 3.1 Study population

This study included 2697 patients diagnosed with a primary SIA. Of all patients, 5.6% were diagnosed with a genetic predisposing syndrome; 107 patients (4.0%) had LS, and 42 patients (1.6%) had a diagnosis of a polyposis syndrome (Table 1). Other underlying predisposing conditions were diagnosed in 6.8% of all SIA patients, of which IBD and celiac disease were diagnosed in 106 patients (3.9%) and 79 patients (2.9%), respectively. In total, four patients (0.2%) had two predisposing conditions. These patients were excluded from further analyses, leaving 326 SIA patients with a single predisposing condition for this study. Patient characteristics are shown in Table 2. Multivariable Cox regression analysis for OS in patients diagnosed with primary SIA showed younger age, earlier stage, distal tumors, surgery of the primary tumor, and a diagnosis of LS as independent factors associated with better OS (Table S1). Within each subgroup of predisposing conditions, earlier stage was associated with better OS (Figure S1A–D).

**TABLE 1:** Diagnoses of predisposing conditions in patients diagnosed with small intestinal adenocarcinoma from January 1999 through December 2019 in the Netherlands.

Diagnoses of predisposing conditions	*n* (%)
Genetic predisposing syndromes	149 (5.6)
Lynch syndrome	107 (4.0)
Polyposis syndrome	42 (1.6)
Familial adenomatous polyposis (FAP)	20 (0.7)
MUTYH‐associated polyposis	7 (0.3)
Peutz–Jeghers syndrome	3 (0.1)
Juvenile polyposis	1 (0.04)
Attenuated FAP	1 (0.04)
Polyposis not otherwise specified	10 (0.4)
Non‐genetic predisposing conditions	185 (6.8)
Inflammatory bowel disease (IBD)	106 (3.9)
Crohn's disease	79 (2.9)
Ulcerative colitis	21 (0.8)
IBD unknown	6 (0.2)
Celiac disease	79 (2.9)

*Note*: The shown percentages are calculated for the total cohort of small intestinal adenocarcinoma patients (*n* = 2697), including four patients with a double diagnosis of a predisposing condition.

Abbreviations: *n*, number of patients.

**TABLE 2:** Characteristics of patient diagnosed with small intestinal adenocarcinoma from 1999 through 2019 in the Netherlands according to predisposing condition. Patients with a double diagnosis of a predisposing condition are excluded from analysis.

		Genetic predisposing syndromes		Non‐genetic predisposing conditions	
	No predisposing condition	Lynch syndrome	Polyposis syndrome	IBD	Celiac disease
	(*n* = 2367)	(*n* = 104)	(*n* = 41)	(*n* = 103)	(*n* = 78)
	*n* (%)	*n* (%)	*n* (%)	*n* (%)	*n* (%)
Sex					
Male	1219 (51.5)	68 (65.4)	23 (56.1)	54 (52.4)	44 (56.4)
Female	1148 (48.5)	36 (34.6)	18 (43.9)	49 (47.6)	34 (43.6)
*p*‐Value*		.007	.669	.933	.460
Age at SIA diagnosis (years)					
Median [IQR]	70 [61–78]	60.5 [50.8–71]	56 [47–65]	59 [51–73]	69.5 [61–74]
<50	173 (7.3)	22 (21.2)	14 (34.2)	21 (20.4)	5 (6.4)
50–59	334 (14.1)	28 (26.9)	11 (26.8)	31 (30.1)	11 (14.1)
60–69	638 (27.0)	20 (19.2)	9 (22.0)	19 (18.4)	23 (29.5)
70–79	738 (31.2)	32 (30.8)	6 (14.6)	23 (22.3)	30 (38.5)
≥80	484 (20.5)	2 (1.9)	1 (24.4)	9 (8.7)	9 (11.5)
*p*‐Value*		<.001	<.001	<.001	.341
Period of diagnosis					
1999–2005	515 (21.8)	15 (14.4)	11 (26.8)	16 (15.5)	18 (23.1)
2006–2012	807 (34.1)	33 (31.7)	12 (29.3)	43 (41.8)	26 (33.3)
2013–2019	1045 (44.2)	56 (53.8)	18 (43.9)	44 (42.7)	34 (43.6)
*p*‐Value*		.092	.687	.173	.962
Tumor location					
Duodenum	1512 (63.9)	51 (49.0)	22 (53.7)	16 (15.5)	30 (38.5)
Jejunum	416 (17.6)	33 (31.7)	11 (26.8)	7 (6.8)	34 (43.6)
Ileum	275 (11.6)	12 (11.5)	4 (9.8)	69 (67.0)	7 (9.0)
NOS	164 (6.9)	8 (7.7)	4 (9.8)	11 (10.7)	7 (9.0)
*p*‐Value*		.002	.333	<.001	<.001
Grade of differentiation					
Well differentiated	119 (5.0)	6 (5.8)	4 (9.8)	12 (11.7)	3 (3.9)
Moderately differentiated	894 (37.8)	57 (54.8)	21 (51.2)	35 (34.0)	27 (34.6)
Poorly differentiated	568 (24.0)	20 (19.2)	6 (14.6)	29 (28.2)	34 (43.6)
Undefined	786 (33.2)	21 (20.2)	10 (24.4)	27 (26.2)	14 (17.9)
*p*‐Value*		.076	.096	.020	.024
TNM stage at diagnosis					
Stage I	127 (5.4)	10 (9.6)	5 (12.2)	13 (12.6)	9 (11.5)
Stage II	598 (25.3)	55 (52.9)	9 (22.0)	42 (40.8)	28 (35.9)
Stage III	595 (25.1)	26 (25.0)	13 (31.7)	25 (24.3)	26 (33.3)
Stage IV	869 (36.7)	13 (12.5)	10 (24.4)	20 (19.4)	13 (16.7)
Undefined	178 (7.5)	0 (0.0)	4 (9.8)	3 (2.9)	2 (2.6)
*p*‐Value*		<.001	.156	<.001	<.001
Site of synchronous metastases^a^					
Liver	458 (57.5)	4 (33.3)	5 (62.5)	11 (55.0)	4 (40.0)
Peritoneum	286 (35.9)	3 (25.0)	2 (25.0)	7 (35.0)	5 (50.0)
Distant lymph node metastases	145 (18.2)	5 (41.7)	1 (12.5)	3 (15.0)	3 (30.0)
Lung and pleura	120 (15.1)	0 (0.0)	1 (12.5)	2 (10.0)	1 (10.0)
Others	300 (37.6)	7 (58.3)	5 (62.5)	6 (30.0)	4 (40.0)
Unknown	72 (8.3)	1 (7.7)	2 (20.0)	0 (0.0)	3 (23.1)
*p*‐Value*		NA	NA	NA	NA
MMR status^b^					
pMMR	395 (84.6)	1 (1.0)	8 (72.7)	28 (84.8)	8 (47.1)
dMMR	72 (15.4)	103 (99.0)	3 (27.3)	5 (15.2)	9 (52.9)
Unknown	1900 (80.3)	0 (0.0)	30 (73.2)	70 (68.0)	61 (78.2)
*p*‐Value*		<.001	.391	1	<.001
Treatment					
Surgery of primary tumor	1244 (52.6)	88 (84.6)	27 (65.9)	81 (78.6)	59 (75.6)
*p*‐Value*		<.001	.125	<.001	<.001
Systemic therapy	546 (23.1)	18 (17.3)	8 (19.5)	23 (22.3)	12 (15.4)
*p*‐Value*		.211	.727	.957	.146
Radiotherapy	64 (2.7)	1 (1.0)	1 (2.4)	1 (0.9)	0 (0.0)
*p*‐Value*		.523	1	.522	<.001
Overall survival (months)					
Median [95% CI]	13.0 [11.8–14.2]	213.1 [99.3–NA]	61.3 [19.7–NA]	29.5 [20.3–69.8]	50.4 [24.6–124.7]
*p*‐value*		<.001	.003	<.001	<.001

Abbreviations: 95% CI, 95% confidence interval; dMMR, mismatch repair deficient; IBD, inflammatory bowel disease; IQR, interquartile range; MMR, mismatch repair; *n*, number of patients; NA, not applicable; NOS, not otherwise specified; pMMR, mismatch repair proficient; SIA, small intestinal adenocarcinoma; TNM, tumor node metastases.

^a^ Percentages are based on patients with registered metastasis.

^b^ Percentages are based on the number of patients with known MMR status.

* The presented *p*‐values refer to comparisons with SIAs with no predisposing condition.

### 3.2 Lynch syndrome

Of LS‐related SIA patients (*n* = 104), 65.4% had the male sex, and the median age at diagnosis was 60.5 years [IQR 50.8–71] (Table 2). Tumors were mainly located in the duodenum (49.0%) and diagnosed at stage II (52.9%). One SIA in an individual with LS was MMR proficient (pMMR) (1.0%).

Compared to sporadic SIA patients, SIAs in patients with LS are more often located in the jejunum (*p* = .002), present in males (*p* = .007), diagnosed at a younger age (*p* < .001), and at a less advanced stage (*p* < .001) (Table 2).

**FIGURE 1:**
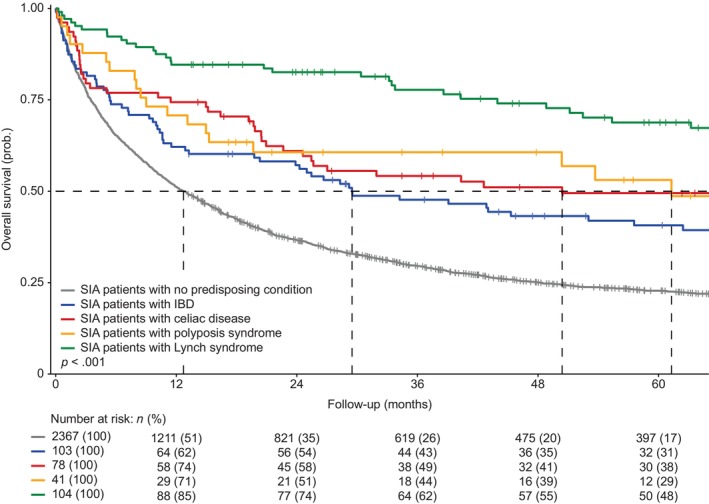
Five‐year overall survival of patient diagnosed with small intestinal adenocarcinoma from 1999 through 2019 in the Netherlands per predisposing condition. IBD, inflammatory bowel disease; *n*, number of patients; prob., probability; SIA, small intestinal adenocarcinoma.

For SIA patients with LS, the median OS (mOS) was 213.1 months (95% CI 99.3–NA), suggesting a survival benefit compared to sporadic SIA patients (Table 2 and Figure 1). Younger age emerged as an independent factor associated with better OS (Table S2).

Data on germline variants of the MMR genes were available in 74.0% of SIA patients with LS. The most common pathogenic variant found was *MSH2* (41.6%), followed by *MLH1* (26.0%), *MSH6* (18.2%), and *PMS2* (14.3%). Patients with a pathogenic variant in *PMS2* were oldest at the time of SIA diagnosis (Table 3).

**TABLE 3:** Characteristics of patients with Lynch syndrome diagnosed with small intestinal adenocarcinoma (SIA) from 1999 through 2019 in the Netherlands according to pathogenic variant in mismatch repair (MMR) gene. Patients with a double diagnosis of a predisposing condition are excluded from analysis.

	Lynch syndrome	*path_MLH1*	*path_MSH2*	*path_MSH6*	*path_PMS2*	*p*‐Value
	(*n* = 104)	(*n* = 20)	(*n* = 32)	(*n* = 14)	(*n* = 11)	
	*n* (%)	*n* (%)	*n* (%)	*n* (%)	*n* (%)	
Sex						
Male	68 (65.4)	13 (65.0)	19 (59.4)	8 (57.1)	6 (54.5)	.926
Female	36 (34.6)	7 (35.0)	13 (40.6)	6 (42.9)	5 (45.5)	
Age at SIA diagnosis (years)						
Median [IQR]	60.5 [50.8–71.0]	60.5 [50.5–68.0]	57.5 [52.0–70.0]	60.5 [50.8–69.3]	68.0 [54.5–72.0]	
<50	22 (21.2)	5 (25.0)	7 (21.9)	2 (14.3)	2 (18.2)	.695
50–59	28 (26.9)	4 (20.0)	12 (37.5)	4 (28.6)	2 (18.2)	
60–69	20 (19.2)	6 (30.0)	4 (12.5)	4 (28.6)	2 (18.2)	
70–79	32 (30.8)	4 (20.0)	8 (25.0)	4 (28.6)	5 (45.5)	
≥80	2 (1.9)	1 (5.0)	1 (3.1)	0 (0.0)	0 (0.0)	
Period of diagnosis						
1999–2005	15 (14.4)	2 (10.0)	8 (25.0)	0 (0.0)	1 (9.1)	.431
2006–2012	33 (31.7)	9 (45.0)	8 (25.0)	6 (42.9)	4 (36.4)	
2013–2019	56 (53.8)	9 (45.0)	16 (50.0)	8 (57.1)	6 (54.5)	
Tumor location						
Duodenum	51 (49.0)	10 (50.0)	15 (46.9)	8 (57.1)	8 (72.7)	.034
Jejunum	33 (31.7)	9 (45.0)	8 (25.0)	2 (14.3)	3 (27.3)	
Ileum	12 (11.5)	0 (0.0)	5 (15.6)	4 (28.6)	0 (0.0)	
NOS	8 (7.7)	1 (5.0)	4 (12.5)	0 (0.0)	0 (0.0)	
Grade of differentiation						
Well differentiated	6 (5.8)	0 (0.0)	3 (9.4)	1 (7.1)	1 (9.1)	<.001
Moderately differentiated	57 (54.8)	13 (65.0)	16 (50.0)	9 (64.3)	5 (45.5)	
Poorly differentiated	20 (19.2)	5 (25.0)	7 (21.9)	0 (0.0)	2 (2.1)	
Undefined	21 (20.2)	2 (10.0)	6 (18.8)	4 (28.6)	3 (27.3)	
TNM stage at diagnosis						
Stage I	10 (9.6)	1 (5.0)	4 (12.5)	1 (7.1)	0 (0.0)	.050
Stage II	55 (52.9)	13 (65.0)	16 (50.0)	7 (50.0)	6 (54.5)	
Stage III	26 (25.0)	4 (20.0)	6 (18.8)	5 (35.7)	4 (36.4)	
Stage IV	13 (12.5)	2 (10.0)	6 (4.2)	1 (7.1)	1 (9.1)	
Undefined	0 (0.0)	0 (0.0)	0 (0.0)	0 (0.0)	0 (0.0)	
Site of synchronous metastases^a^						
Liver	4 (30.8)	1 (50.0)	1 (20.0)	0 (0.0)	0 (0.0)	NA
Peritoneum	3 (23.1)	1 (50.0)	2 (40.0)	0 (0.0)	0 (0.0)	
Distant lymph node metastasis	5 (38.5)	0 (0.0)	0 (0.0)	1 (100.0)	1 (100.0)	
Lung and pleura	0 (0.0)	0 (0.0)	0 (0.0)	0 (0.0)	0 (0.0)	
Others	7 (53.8)	0 (0.0)	2 (40.0)	1 (100.0)	1 (100.0)	
Unknown	0 (0.0)	0 (0.0)	1 (16.7)	0 (0.0)	0 (0.0)	
MMR status^b^						
pMMR	1 (1.0)	0 (0.0)	0 (0.0)	1 (7.1)	0 (0.0)	.025
dMMR	103 (99.0)	20 (100.0)	32 (100.0)	13 (92.9)	11 (100.0)	
Unknown	0 (0.0)	0 (0.0)	0 (0.0)	0 (0.0)	0 (0.0)	
Treatment						
Surgery of primary tumor	88 (84.6)	15 (75.0)	27 (84.4)	11 (78.6)	9 (81.8)	.873
Systemic therapy	18 (17.3)	1 (5.0)	6 (18.8)	2 (14.3)	3 (27.3)	.365
Radiotherapy	1 (1.0)	0 (0.0)	1 (3.1)	0 (0.0)	0 (0.0)	<.001
Overall survival (months)						
Median [95% CI]	213.1 [99.3–NA]	NA [55.4–NA]	190.8 [63.2–NA]	NA [51.2–NA]	84.0 [48.8–NA]	.676

Abbreviations: *n*, number of patients; IQR, interquartile range; NOS, not otherwise specified; 95% CI, 95% confidence interval; TNM, tumor node metastases; NA, not applicable; MMR, mismatch repair; pMMR, mismatch repair proficient; dMMR, mismatch repair deficient.

^a^ Percentages are based on patients with registered metastasis.

^b^ Percentages are based on the number of patients with known MMR status.

### 3.3 Polyposis syndromes

SIA patients with a diagnosis of polyposis syndrome (*n* = 41) had a median age of 56 years [IQR 47–65]. Compared to sporadic SIA, the median age at diagnosis was significantly lower in polyposis patients (*p* < .001) (Table 2). No significant difference was found in tumor stage between SIA patients with polyposis compared to sporadic SIA patients.

The mOS of SIA patients with polyposis syndrome was 61.3 months (95% CI 19.7–NA), indicating a better outcome compared to sporadic SIA patients (*p* = .003) (Table 2 and Figure 1). Only early stage was independently associated with better OS (Table S3).

Among the 41 SIA patients with polyposis, 19 had FAP, seven had a MAP diagnosis, three were diagnosed with PJS, and 10 were classified as polyposis not otherwise specified (Table 4). For both FAP and PJS, a male predominance was found (68.4% and 66.7%, respectively), in contrast to MAP (28.6%). The most common tumor location was the duodenum, followed by the jejunum for FAP (42.1% and 31.6%, respectively) and MAP (71.4% and 28.6%, respectively). In FAP patients, three SIAs were located in the ileum, of whom two were found in an ileostomy and one in an ileoanal pouch. For PJS, SIAs were dominantly located in the jejunum (66.7%). MMR status was only determined in seven tumors of FAP and four tumors of MAP patients. Of these, two tumors for FAP and one tumor for MAP were MMR deficient (dMMR). Between the different polyposis syndromes, no significant differences in tumor characteristics were found, but admittedly, numbers are small in this patient group (Table 4).

**TABLE 4:** Characteristics of patients with a polyposis syndrome diagnosed with small intestinal adenocarcinoma (SIA) from 1999 through 2019 in the Netherlands according to specific polyposis syndrome. Patients with a double diagnosis of a predisposing condition are excluded from analysis.

	Polyposis syndrome	FAP	MAP	PJS	Polyposis NOS	*p*‐Value
	(*n* = 41)	(*n* = 19)	(*n* = 7)	(*n* = 3)	(*n* = 10)	
	*n* (%)	*n* (%)	*n* (%)	*n* (%)	*n* (%)	
Sex						
Male	23 (56.1)	13 (68.4)	2 (28.6)	2 (66.7)	5 (50.0)	.332
Female	18 (43.9)	6 (31.6)	5 (71.4)	1 (33.3)	5 (50.0)	
Age at SIA diagnosis (years)						
Median [IQR]	56 [47–65]	56 [49.0–65.0]	56 [52.5–72.0]	43 [42.0–46.5]	60.5 [51.3–67.5]	
<50	14 (34.2)	6 (31.6)	1 (14.3)	2 (66.7)	3 (30.0)	.999
50–59	11 (26.8)	5 (26.3)	3 (42.9)	1 (33.3)	2 (20.0)	
60–69	9 (22.0)	5 (26.3)	1 (14.3)	0 (0.0)	3 (30.0)	
70–79	6 (14.6)	3 (15.8)	1 (14.3)	0 (0.0)	2 (20.0)	
≥80	1 (24.4)	0 (0.0)	1 (14.3)	0 (0.0)	0 (0.0)	
Period of diagnosis						
1999–2005	11 (26.8)	3 (15.8)	0 (0.0)	2 (66.7)	6 (60.0)	.071
2006–2012	12 (29.3)	7 (36.8)	1 (14.3)	0 (0.0)	2 (20.0)	
2013–2019	18 (43.9)	9 (47.4)	6 (85.7)	1 (33.3)	2 (20.0)	
Tumor location						
Duodenum	22 (53.7)	8 (42.1)	5 (71.4)	1 (33.3)	6 (60.0)	.689
Jejunum	11 (26.8)	6 (31.6)	2 (28.6)	2 (66.7)	1 (10.0)	
Ileum	4 (9.8)	3 (15.8)	0 (0.0)	0 (0.0)	1 (10.0)	
NOS	4 (9.8)	2 (10.5)	0 (0.0)	0 (0.0)	2 (20.0)	
Grade of differentiation						
Well differentiated	4 (9.8)	1 (5.3)	0 (0.0)	2 (66.7)	1 (10.0)	.150
Moderately differentiated	21 (51.2)	10 (52.6)	4 (57.1)	1 (33.3)	5 (50.0)	
Poorly differentiated	6 (14.6)	3 (15.8)	2 (28.6)	0 (0.0)	0 (0.0)	
Undefined	10 (24.4)	5 (26.3)	1 (14.3)	0 (0.0)	4 (40.0)	
TNM stage at diagnosis						
Stage I	5 (12.2)	4 (21.1)	0 (0.0)	0 (0.0)	0 (0.0)	.991
Stage II	9 (22.0)	4 (21.1)	2 (28.6)	1 (33.3)	2 (20.0)	
Stage III	13 (31.7)	5 (26.3)	3 (42.9)	2 (66.7)	3 (30.0)	
Stage IV	10 (24.4)	5 (26.3)	2 (28.6)	0 (0.0)	2 (20.0)	
Undefined	4 (9.8)	0 (5.3)	0 (0.0)	0 (0.0)	3 (30.0)	
Site of synchronous metastases^a^						
Liver	5 (62.5)	3 (75.0)	0 (0.0)	/	1 (100)	NA
Peritoneum	2 (25.0)	0 (0.0)	1 (50.0)	/	0 (0.0)	
Distant lymph node metastasis	1 (12.5)	0 (0.0)	1 (50.0)	/	0 (0.0)	
Lung and pleura	1 (12.5)	1 (25.0)	0 (0.0)	/	0 (0.0)	
Others	5 (62.5)	2 (50.0)	1 (50.0)	/	1 (100)	
Unknown	2 (20.0)	1 (20.0)	0 (0.0)	/	1 (50.0)	
MMR status^b^						
pMMR	8 (72.7)	5 (71.4)	3 (75.0)	/	/	1
dMMR	3 (27.3)	2 (28.6)	1 (25.0)	/	/	
Unknown	30 (73.2)	12 (63.2)	3 (42.9)	3 (100)	10 (100)	
Treatment						
Surgery of primary tumor	27 (65.9)	12 (63.2)	5 (71.4)	3 (100)	6 (60.0)	.908
Systemic therapy	8 (19.5)	4 (21.1)	2 (28.6)	0 (0.0)	1 (10.0)	.326
Radiotherapy	1 (2.4)	0 (0.0)	0 (0.0)	3 (100)	1 (10.0)	.129
Overall survival (months)						
Median [95% CI]	61.3 [19.7–NA]	71.9 [53.7–NA]	50.4 [5.36–NA]	NA [15.3–NA]	10.8 [5.0–NA]	.261

Abbreviations: 95% CI, 95% confidence interval; dMMR, mismatch repair deficient; FAP, familial adenomatous polyposis; IQR, interquartile range; MAP, MUTYH‐associated polyposis; MMR, mismatch repair; *n*, number of patients; NA, not applicable; NOS, not otherwise specified; PJS, Peutz–Jeghers polyposis; pMMR, mismatch repair proficient; TNM, tumor node metastases.

^a^ Percentages are based on patients with registered metastasis.

^b^ Percentages are based on the number of patients with known MMR status.

### 3.4 Inflammatory bowel disease

SIA patients with IBD (*n* = 103) had a median age of 59 years [IQR 51–73] at diagnosis, and tumors were most frequently diagnosed at stage II (40.8%) (Table 2). When compared to sporadic SIAs, SIAs of IBD patients were more often found at a younger age (*p* < .001) and earlier stage (*p* < .001), located in the ileum (*p* < .001), and more frequently surgically resected (*p* < .001), (Table 2).

Of SIA patients with IBD, most patients were diagnosed with CD (*n* = 78), followed by UC (*n* = 19) and IBD‐U (*n* = 6) (Table 5). Tumors were most frequently located in the duodenum for UC (63.2%) and in the ileum for CD and IBD‐U (78.2% and 50.0%, respectively) (*p* < .001). Of the ileum‐located SIAs (*n* = 5) in UC patients, two originated from the pouch and one from the stoma. SIAs of patients with UC were more often diagnosed in an advanced stage than patients with CD or IBD‐U (*p* = .016), and surgery of the primary tumor was performed in 84.6% for CD, 63.2% for UC, and 50.0% for IBD‐U (*p* = .021) (Table 5).

**TABLE 5:** Characteristics of patients with inflammatory bowel disease (IBD) diagnosed with small intestinal adenocarcinoma (SIA) from 1999 through 2019 in the Netherlands according to specific IBD diagnosis. Patients with a double diagnosis of a predisposing condition are excluded from analysis.

	IBD	Crohn's disease	Ulcerative colitis	IBD unknown	*p*‐Value
	(*n* = 103)	(*n* = 78)	(*n* = 19)	(*n* = 6)	
	*n* (%)	*n* (%)	*n* (%)	*n* (%)	
Sex					
Male	54 (52.4)	40 (51.3)	12 (63.2)	2 (33.3)	.408
Female	49 (47.6)	38 (48.7)	7 (36.8)	4 (66.7)	
Age at SIA diagnosis (years)					
Median [IQR]	59 [51–73]	58 [51–71]	62 [55.5–73.0]	68 [49.5–85.75]	
<50	21 (20.4)	15 (19.2)	4 (21.1)	2 (33.3)	.621
50–59	31 (30.1)	27 (34.6)	3 (15.8)	1 (16.7)	
60–69	19 (18.4)	14 (18.0)	5 (26.3)	0 (0.0)	
70–79	23 (22.3)	17 (21.8)	6 (31.6)	0 (0.0)	
≥80	9 (8.7)	5 (6.4)	1 (5.3)	3 (50.0)	
Period of diagnosis					
1999–2005	16 (15.5)	11 (14.1)	4 (21.1)	1 (16.7)	.804
2006–2012	43 (41.8)	35 (44.9)	6 (31.6)	2 (33.3)	
2013–2019	44 (42.7)	32 (41.0)	9 (47.4)	3 (50.0)	
Tumor location					
Duodenum	16 (15.5)	3 (3.8)	12 (63.2)	1 (16.7)	<.001
Jejunum	7 (6.8)	6 (7.7)	0 (0.0)	1 (16.7)	
Ileum	69 (67.0)	61 (78.2)	5 (26.3)	3 (50.0)	
NOS	11 (10.7)	8 (10.3)	2 (10.5)	1 (16.7)	
Grade of differentiation					
Well differentiated	12 (11.7)	10 (12.8)	1 (5.3)	1 (16.7)	.734
Moderately differentiated	35 (34.0)	29 (37.2)	4 (21.1)	2 (33.3)	
Poorly differentiated	29 (28.2)	22 (28.2)	6 (31.6)	1 (16.7)	
Undefined	27 (26.2)	17 (21.8)	8 (42.1)	2 (33.3)	
TNM stage at diagnosis					
Stage I	13 (12.6)	9 (11.5)	3 (15.8)	1 (16.7)	.016
Stage II	42 (40.8)	38 (48.7)	2 (10.5)	2 (33.3)	
Stage III	25 (24.3)	18 (23.1)	6 (31.6)	1 (16.7)	
Stage IV	20 (19.4)	12 (15.4)	7 (36.8)	1 (16.7)	
Undefined	3 (2.9)	1 (1.3)	1 (5.3)	1 (16.7)	
Site of synchronous metastases^a^					
Liver	11 (55.0)	6 (50.0)	4 (57.1)	1 (100.0)	NA
Peritoneum	7 (35.0)	5 (41.7)	2 (28.5)	0 (0.0)	
Distant lymph node metastasis	3 (15.0)	0 (0.0)	3 (42.9)	0 (0.0)	
Lung and pleura	2 (10.0)	0 (0.0)	2 (28.5)	0 (0.0)	
Others	6 (30.0)	2 (16.7)	4 (57.1)	0 (0.0)	
Unknown	0 (0.0)	0 (0.0)	0 (0.0)	0 (0.0)	
MMR status^b^					
pMMR	28 (84.8)	24 (88.9)	2 (50.0)	2 (100)	.054
dMMR	5 (15.2)	3 (11.11)	2 (50.0)	0 (0.0)	
Unknown	70 (68.0)	51 (65.4)	15 (78.9)	4 (66.7)	
Treatment					
Surgery of primary tumor	81 (78.6)	66 (84.6)	12 (63.2)	3 (50.0)	.021
Systemic therapy	23 (22.3)	13 (16.7)	8 (42.1)	2 (33.3)	.407
Radiotherapy	1 (0.9)	0 (0.0)	1 (5.3)	0 (0.0)	.001
Overall survival (months)					
Median [95% CI]	29.5 [20.3–69.8]	53.0 [26.7–155.3]	9.3 [1.6–45.3]	23.8 [3.1–NA]	.023

Abbreviations: 95% CI, 95% confidence interval; dMMR, mismatch repair deficient; IQR, interquartile range; MMR, mismatch repair; *n*, number of patients; NA, not applicable; NOS, not otherwise specified; pMMR, mismatch repair proficient; TNM, tumor node metastases.

^a^ Percentages are based on patients with registered metastasis.

^b^ Percentages are based on the number of patients with known MMR status.

The mOS for IBD‐related SIA patients was 29.5 months (95% CI 20.3–69.8), demonstrating a more favorable outcome compared to sporadic SIA patients (*p* < .001) (Table 2 and Figure 1). A younger age, as well as an earlier stage, was independently associated with better OS (Table S4). Within IBD‐related SIA, the mOS was best for CD (53.0 months, 95% CI 26.7–155.3), followed by IBD‐U (28.3 months, 95% CI 3.1–NA) and UC (9.3 months, 95% CI 1.6–45.3) (*p* = .020) (Table 5).

### 3.5 Celiac disease

In celiac‐related SIA patients (*n* = 78), the median age at diagnosis was 69.5 years [IQR 61–74], and tumors were mainly diagnosed at stage II (35.9%), followed by stage III (33.3%) (Table 2). MMR status was determined in 17 tumors (21.8%), of which nine were dMMR.

Celiac‐related SIAs were more often located in the jejunum (*p* < .001), diagnosed at an earlier stage (*p* < .001) and surgically resected (*p* < .001), compared to sporadic SIAs (Table 2).

The mOS of celiac‐related SIA patients was 50.4 months (95% CI 24.6–124.7), suggesting a survival benefit compared to sporadic SIA (*p* < .001) (Table 2 and Figure 1). A younger age, jejunal tumors, and surgery of the primary tumor were identified as factors independently associated with better OS (Table S5).

## 4 DISCUSSION

This nationwide retrospective study evaluating predisposing conditions in 2697 SIA patients represents, to our knowledge, the largest analysis to date on this topic. We found a genetic predisposing syndrome in 5.6% of SIA patients: 4.0% for LS and 1.6% for a polyposis syndrome, including 0.7% for FAP. Non‐genetic predisposing conditions were found in an additional 6.8%, with prevalences of 0.8% for UC and 2.9% for both CD and celiac disease. These prevalence rates for both genetic and non‐genetic predisposing conditions align with ranges found in previous studies varying from 1.0% to 9.3% and from 0.5% to 10.4%, respectively.^8^, ^15^ The differences in prevalence may arise from relatively small cohorts of 205 and 346 patients and the limited selection of included predisposing conditions in the latter studies.^8^, ^15^ Hence, the nationwide design of our study provides a more robust prevalence rate estimation. Additionally, Asian countries seem to have a lower fraction of adenocarcinomas within small intestinal cancer subtypes compared to Western countries.^21^ Our findings suggest a higher prevalence of predisposing conditions in SIA as compared to CRC. Of CRC cases, LS and FAP account for 1.7% and 0.03%–0.09%, while CD and UC represent 0.4% and 0.8%, respectively.^16^, ^17^, ^18^ These prevalence differences support the existing evidence that SIA is a unique malignancy, requiring tailored treatment protocols and dedicated trials specifically addressing SIA.

Several genetic and non‐genetic conditions seem to increase the risk of developing SIA.^14^ Therefore, these patients might benefit from small bowel neoplasia screening. The lifetime SIA risk in LS is 4%, which is over 100 times higher than in the general population.^22^, ^23^ This risk closely resembles the 4.1% lifetime risk of CRC in the general population, warranting population‐based screening recommendations.^24^ Thus far, gastro‐duodenal endoscopy is only included in surveillance for polyposis patients.^25^, ^26^ Additionally, visualization of the jejunum and ileum remains challenging.^27^ New techniques like video capsule endoscopy, double‐balloon enteroscopy, and MR enteroclysis have sparked debate about including small bowel neoplasia screening for at‐risk populations, especially in LS.^27^ Lack of specific incidence rates and proven benefit of screening in these populations complicates this debate. Extra awareness for cancer symptoms and during small bowel examination in these patients is necessary.

Of all SIAs, a pMMR phenotype was mostly found in patients with IBD and polyposis, conforming to the literature for FAP.^28^ Patients with LS or celiac disease often presented with dMMR SIAs, as previously described.^29^ Notably, in this cohort, MMR status was determined in less than 20% of SIAs across all subgroups, with MMR testing not routinely conducted in SIA during the study period; selection bias might be present. Despite this, the relatively high incidence of dMMR SIAs (52.9%) in celiac disease corresponds with literature.^29^ This might be of positive influence on OS in these patients, although no differences at the chromosomal level have been reported between celiac‐related and sporadic SIA.^30^ Clinicians should consider celiac disease in dMMR SIAs, in addition to LS. Overall, more studies into MMR status in SIA patients are warranted.

Both genetic and non‐genetic predisposing conditions were associated with improved survival as compared to sporadic SIA in our cohort. This is in contrast to another study showing predisposing conditions to be associated with worse OS.^31^ This difference is probably related to small numbers of patients with predisposing conditions (*n* = 35 for local disease, *n* = 11 for metastatic disease) in the study by Aparicio et al.^31^ We propose that the improved survival associated with a diagnosis of a predisposing condition in this nationwide study may partly be explained by earlier detection and potential different tumor biology. This might reflect both patient and clinician vigilance for specific manifestations in these cohorts, as well as surveillance in specifically polyposis patients. To further investigate this, more data is needed on the indication of diagnosis (whether patients presented with symptoms or tumor were diagnosed by surveillance) and differences in molecular mechanisms.

### 4.1 Genetic predisposing syndromes

In our cohort LS, was the most common predisposing condition.^17^ SIAs of LS patients were more often diagnosed in the male sex and at a young age (median 60.5 years), conforming to other SIA studies and as seen in LS‐associated CRCs.^15^, ^32^, ^33^, ^34^

For SIA patients, LS was associated with better OS compared to other predisposing conditions and sporadic cases, confirming previous observations.^15^ This may be explained by distinct tumor biology, particularly microsatellite instability, which is associated with lower metastatic potential and better immunotherapeutic responses.^35^, ^36^

In this SIA cohort, germline mutations in *MSH2* were most frequently observed, followed by *MLH1*. This high‐risk profile for *MLH1* and *MSH2* carriers compared to *MSH6* and *PMS2* carriers aligns with previous literature and with data for CRC.^34^, ^37^, ^38^ However, a higher risk for *MLH1* compared with *MSH2* carriers for duodenal and small bowel cancer was reported by Møller et al. and Bondana et al., respectively.^38^ These studies often did not distinguish between gastric and small intestinal cancers or only included duodenal SIAs, suggesting a potential different risk profile for SIA specifically in *MSH2* and *MLH1* carriers compared to other upper gastrointestinal cancers. Based on our findings, small bowel endoscopic surveillance in *MSH2* and *MLH1* carriers could be considered, as previously suggested.^34^, ^37^, ^38^ Nevertheless, future research is needed to investigate the impact on prognosis and cost‐effectiveness of such surveillance in these patients.

Of SIA patients with polyposis, the majority had a diagnosis of FAP, followed by MAP. For FAP, this prevalence corresponds with other studies (0.49%–2.0%).^8^, ^15^, ^39^

Among all SIA patient subgroups, polyposis patients were the youngest at diagnosis, and nearly 15 years younger than sporadic SIA patients. The duodenum was mostly affected in FAP and MAP, conforming to earlier literature.^15^ Both the early onset and preferred tumor location can possibly be attributed to the predisposition of multiple duodenal and ampullary polyps at a young age in FAP patients (65%–90%) and to a lesser extent in MAP patients (21.1%), making it more prone for tumor development.^40^, ^41^, ^42^ Moreover, it is postulated that high exposure to undiluted dietary carcinogens and bile acids, primarily in the distal duodenum, contributes to the dysplastic transformation of the existing polyps.^11^, ^43^ It is suggested that the increased risk for jejunal polyps and SIA correlates to the severity of duodenal polyposis.^27^, ^41^ To reduce this risk, guidelines recommend gastroduodenoscopic surveillance with polypectomy for both FAP and MAP from the age of 25.^25^, ^26^ In case of severe duodenal polyposis, prophylactic polypectomies or pancreatoduodenectomies are conducted in FAP patients, which might underestimate the lifetime SIA risk in this group.^43^ Despite these prophylactic interventions, polyps still develop in ileostomas and ileoanal pouches constructed after proctocolectomy.^40^, ^42^ Notably, among the ileal SIAs in our cohort, 50% originated from the stoma and 25% from the pouch. However, it highlights the importance of regular postoperative surveillance in the remaining intestinal part, as stated in the literature.^44^, ^45^ Despite targeted surveillance, SIAs are not found at an earlier stage in polyposis patients compared to sporadic cases, which is in contrast to other predisposing conditions in our cohort. This discrepancy may be due to the high number of duodenal and jejunal polyps in FAP and MAP patients, together with difficult endoscopic visualization for the jejunum and ileum.

The longer survival of SIA patients with polyposis compared to sporadic SIA patients is conform the literature.^15^ Nevertheless, small intestinal cancer is the second leading cause of death in FAP. This, together with our data showing no difference in earlier stage compared to sporadic SIA and the prevalence of SIAs in the stoma and pouch, highlights the importance of regular endoscopic surveillance and the need for dedicated research on risk factors for tumor development in these patients.

### 4.2 Non‐genetic predisposing conditions

Our study showed IBD‐related SIA to be diagnosed at a significantly younger age, at an earlier stage, and more often affecting the ileum compared to sporadic SIA. The younger age at diagnosis is coherent with literature.^16^, ^46^

UC‐related SIA is more likely to follow the distribution of sporadic SIA with the duodenum as the most common tumor location. This might suggest, since the disease mostly affects the colon, that UC has less effect on tumor location in the small intestine. Another suggestion might be clinical misdiagnosis of UC. However, only confirmed diagnoses of predisposing conditions were used in this study, limiting chances of clinical misdiagnosis of predisposing conditions. Therefore, it is not expected to affect these conclusions. A study performed in the United States showed clinical misdiagnosis of UC to be limited to 5.3%; numbers of misdiagnosis in the Netherlands are currently unknown.^47^ Two tumors originated from the pouch and one from the stoma. This questions the rightful origin of the small intestine, as possible colonic or neorectal transformation of the pouch or stoma is previously described.^48^, ^49^ Following CD biology, the ileum is the most commonly affected site in CD, and the ileum appeared to be prone to CD‐related SIAs, as previously published.^46^, ^50^

The prevalence of celiac disease in the Netherlands is 1%. Our reported incidence of celiac disease in SIA patients of 2.9% shows a threefold increase, which is in line with reported incidences of 1.7% and 3%.^14^, ^39^

Our results show that celiac‐related SIAs are predominantly located in the jejunum and mostly diagnosed with local disease, as previously reported.^51^ This may be related to the disease biology with long‐term inflammation, especially in the jejunum.^52^ Furthermore, a less aggressive behavior in celiac‐related SIA compared to sporadic SIA was suggested.^53^

### 4.3 Limitations

The retrospective and non‐randomized nature of this study may have introduced selection bias in received treatment analyses and missing data bias, particularly concerning MMR status, mutational data, and differentiation grade. However, this reflects clinical practice and highlights the need for consensus measures regarding pathologic reports, including MMR and mutation analyses, preferably in national and international guidelines. Furthermore, given the retrospective nature of this study, there is a risk of missed diagnosis of predisposing conditions, especially non‐genetic predisposing conditions, which may result in an underestimation of their prevalence. Nevertheless, this further underscores the importance of identifying predisposing conditions in SIA. No information on the cause of death, cancer progression, and disease progression was available, precluding the calculation of disease‐free, cancer‐specific, and relative survival. These limitations warrant careful interpretation of the results. Furthermore, the relatively small numbers per subgroup limit the power of our conclusions, particularly for factors influencing OS and the possibility to stratify survival analyses.

## 5 CONCLUSION

To the best of our knowledge, this is the most extensive nationwide cohort study describing predisposing conditions in SIA to date and provides a unique overview of the incidence of predisposing conditions in SIA and their influence on both clinical and tumor features over two decades. This study underscores the importance of awareness and tailored surveillance in at‐risk populations leading to earlier detection and improved prognosis. Future research should focus on molecular mechanisms influencing these different tumor characteristics and the role of preventive diagnostics in improving outcome.

## AUTHOR CONTRIBUTIONS

**Jasmijn D. G. Linssen:** Conceptualization; methodology; software; validation; formal analysis; investigation; data curation; writing – original draft; visualization. **Pascale J. M. Schafrat:** Conceptualization; investigation; writing – original draft; methodology; validation; visualization; software; formal analysis; data curation. **Tim R. de Back:** Methodology; validation; resources; writing – review and editing; supervision. **Felice N. van Erning:** Methodology; validation; writing – review and editing; resources; supervision. **Monique E. van Leerdam:** Resources; writing – review and editing; supervision. **Evelien Dekker:** Conceptualization; methodology; validation; writing – review and editing; resources; supervision; project administration. **Louis Vermeulen:** Writing – review and editing; supervision; conceptualization; methodology; validation; resources; project administration; funding acquisition. **Ignace H. J. T. de Hingh:** Conceptualization; methodology; validation; writing – review and editing; resources; supervision; project administration; funding acquisition. **Dirkje W. Sommeijer:** Conceptualization; funding acquisition; methodology; validation; writing – review and editing; project administration; resources; supervision.

## FUNDING INFORMATION

This work is supported by Oncode and The New York Stem Cell Foundation (NYSCF‐I‐R43), and grants from the European Research Council (ERC‐CoG 101045612—NIMICRY), ZonMw (Vici 09‐15018‐21‐10029), and Dutch Cancer Society (KWF 14182). Funder had no role in study design or manuscript.

## CONFLICT OF INTEREST STATEMENT

Louis Vermeulen received consultancy fees from Bayer, MSD, Genentech, Servier, and Pierre Fabre, but these had no relation to the content of this publication. Louis Vermeulen is currently an employee of Genentech Inc. and a shareholder of Roche. Ignace H. J. T. de Hingh received research grants from ROCHE and RanD Biotech, but these had no relation to the content of this publication. Evelien Dekker has endoscopic equipment on a loan from FujiFilm and has received a research grant from FujiFilm. Evelien Dekker received an honorarium for a consultancy from Olympus, Fujifilm, Ambu, InterVenn, Norgine, and Exact Sciences, and speakers' fees from Olympus, GI Supply, Norgine, IPSEN/Mayoly, FujiFilm, and Steris. These had no relationship to the content of this publication. All remaining authors have declared no conflicts of interest.

## ETHICS STATEMENT

This study (number K21.033) was approved by the privacy review board of the Netherlands Cancer Registry. As only pseudonymized data of the Dutch National Cancer Registry were used in this study, informed consent was not required. The study was conducted in accordance with the Declaration of Helsinki.

## Supporting information


**Data S1.** Supporting Information.

## Data Availability

The data that support the findings of this study are available from the corresponding author upon reasonable request.
