# Supplementary material for: Predisposing conditions in patients with small intestinal adenocarcinomas in the Netherlands: A 20‐year nationwide cohort study
Source: Int J Cancer. 2025 Feb 5;157(2):218–31. doi: 10.1002/ijc.35354 (PMC12079630; doi:10.1002/ijc.35354)
Supplement: Supplementary file 1 — Data S1. Supporting Information. [file IJC-157-218-s001.pdf]

Supplementary Information

Predisposing conditions in patients with small intestinal adenocarcinomas in the Netherlands: a 20-year nationwide cohort study.

Jasmijn D.G. Linssen, Pascale J.M. Schafrat, Tim R. de Back, Felice N. van Erning, Monique E. van Leerdam, Evelien Dekker, Louis Vermeulen, Ignace H.J.T. de Hingh & Dirkje W. Sommeijer

Table of Contents:

Table S1. Univariable and multivariable Cox proportional hazard models for overall survival for patients diagnosed with SIA from January 1999 through December 2019 in the Netherlands.

Table S2. Univariable and multivariable Cox proportional hazard models for overall survival for patients with Lynch syndrome diagnosed with SIA from January 1999 through December 2019 in the Netherlands.

Table S3. Univariable and multivariable Cox proportional hazard models for overall survival for patients with a polyposis syndrome diagnosed with SIA from January 1999 through December 2019 in the Netherlands.

Table S4. Univariable and multivariable Cox proportional hazard models for overall survival for patients with IBD diagnosed with SIA from January 1999 through December 2019 in the Netherlands.

Table S5. Univariable and multivariable Cox proportional hazard models for overall survival for patients with celiac disease diagnosed with SIA from January 1999 through December 2019 in the Netherlands.

Figure S1. Five-year overall survival per stage of disease of patients diagnosed with SIA and a diagnosis of (A) Lynch syndrome, (B) polyposis syndrome, (C) inflammatory bowel syndrome and (D) celiac disease. n, number of patients; Prob., probability.

Table S1. Univariable and multivariable Cox proportional hazard models for overall survival for patients diagnosed with SIA from January 1999 through December 2019 in the Netherlands.

	Univariable			Multivariable		
	n	HR (95% CI)	*p*-value	n	HR (95% CI)	*p*-value
Age, years[a]	2693	1.03 (1.03 – 1.03)	<0.001	2506	1.03 (1.02 – 1.03)	<0.001
Sex						
Male	1408	1.00 (reference)	0.904			
Female	1285	1.01 (0.92 – 1.10)				
Period of diagnosis						
1999-2005	575	1.00 (reference)	0.691			
2006-2012	921	0.95 (0.85 – 1.07)				
2013-2019	1997	0.961 (0.86 – 1.08)				
Tumor location						
Duodenum	1631	1.00 (reference)	<0.001	1465	1.00 (reference)	<0.001
Jejunum	501	0.55 (0.48 – 0.62)		497	0.75 (0.66 – 0.85)	
Ileum	367	0.62 (0.55 – 0.71)		363	0.91 (0.78 – 1.05)	
NOS	194	1.03 (0.88 – 1.21)		181	1.08 (0.91 – 1.28)	
TNM Stage at diagnosis						
Stage I	164	0.19 (0.156 – 0.24)	<0.001	164	0.25 (0.20 – 0.31)	<0.001
Stage II	732	0.23 (0.20 – 0.26)		732	0.33 (0.29 – 0.37)	
Stage III	685	0.35 (0.32 – 0.40)		685	0.54 (0.47 – 0.61)	
Stage IV	925	1.00 (reference)		925	1.00 (reference)	
Missing cases	187					
Predisposing condition						
No	2367	1.00 (reference)	<0.001	2189	1.00 (reference)	<0.001
Lynch syndrome	104	0.27 (0.19 – 0.37)		104	0.46 (0.33 – 0.63)	
Polyposis syndrome	41	0.54 (0.36 – 0.81)		37	0.78 (0.50 – 1.20)	
IBD	103	0.62 (0.48 – 0.79)		100	1.06 (0.81 – 1.38)	
Celiac disease	78	0.55 (0.41 – 0.73)		76	0.77 (0.58 – 1.03)	

Surgery of the primary tumor						
No	1194	1.00 (reference)	<0.001	1022	1.00 (reference)	<0.001
Yes	1499	0.26 (0.24 – 0.29)		1484	0.40 (0.36 – 0.45)	
Systemic therapy						
No	2086	1.00 (reference)	0.357			
Yes	607	0.95 (0.86 – 1.06)				
Radiotherapy						
No	2626	1.00 (reference)	0.003	2453	1.00 (reference)	0.235
Yes	67	1.45 (1.13 – 1.86)		53	0.84 (0.63 – 1.12)	
MMR status						
pMMR	440	1.00 (reference)	<0.001			
dMMR	192	0.38 (0.29 – 0.49)				
Missing cases	2061					
Grade of differentiation						
Well differentiated	144	1.00 (reference)				
Moderately differentiated	1034	1.23 (0.99 – 1.53)	<0.001			
Poorly differentiated	657	1.70 (1.36 – 2.13)				
Missing cases	858					
Mucinous differentiation						
No	117	1.00 (reference)				
Yes, < 50%	242	1.17 (0.90 – 1.52)	0.397			
Yes, > 50%	216	1.03 (0.79 – 1.35)				
Missing cases	2118					
Lymphangioinvasion						
No	473	1.00 (reference)	<0.001			
Yes	512	2.01 (1.71 – 2.37)				
Missing cases	1708					
Perineural invasion						
No	262	1.00 (reference)	<0.001			
Yes	208	1.84 (1.44 – 2.34)				

Missing cases	2223

Univariable Cox regression analysis included 2693 patients. Multivariable Cox regression analysis included 2506 patients, 187 patients excluded due to missings.

[a] Analyzed as a continuous variable.

n, number of patients; HR, hazard ratio; 95% CI, 95% confidence interval; NOS, not otherwise specified; IBD, inflammatory bowel disease; pMMR, mismatch repair proficient; dMMR, mismatch repair deficient.

Table S2. Univariable and multivariable Cox proportional hazard models for overall survival for patients with Lynch syndrome diagnosed with SIA from January 1999 through December 2019 in the Netherlands.

	Univariable			Multivariable		
	n	HR (95% CI)	*p*-value	n	HR (95% CI)	*p*-value
Age, years[a]	104	1.06 (1.02 – 1.09)	<0.001	104	1.06 (1.02 – 1.09)	<0.001
Sex						
Male	68	1.00 (reference)	0.658			
Female	36	0.66 (0.44 – 1.69)				
Period of diagnosis						
1999-2005	15	1.00 (reference)				
2006-2012	33	2.62 (0.75 – 9.15)	0.082			
2013-2019	56	4.10 (1.16 – 14.56)				
Tumor location						
Duodenum	51	1.00 (reference)				
Jejunum	33	1.01 (0.50 – 2.02)	0.454			
Ileum	12	0.54 (0.16 – 1.83)				
NOS	8	0.27 (0.04 – 1.98)				
TNM Stage at diagnosis						
Stage I	10	0.44 (0.14 – 1.43)		10	0.40 (0.12 – 1.42)	
Stage II	55	0.26 (0.10 – 0.65)	0.022	55	0.30 (0.10 – 0.86)	0.170
Stage III	26	0.24 (0.09 – 0.69)		26	0.39 (0.12 – 1.33)	
Stage IV	13	1.00 (reference)		13	1.00 (reference)	
Missing cases	0					
Surgery of the primary tumor						
No	16	1.00 (reference)	0.003	16	1.00 (reference)	0.166
Yes	88	0.33 (0.16 – 0.68)		88	0.54 (0.22 – 1.29)	
Systemic therapy						
No	86	1.00 (reference)	0.307			
Yes	18	1.54 (0.67 – 3.56)				

Radiotherapy			
No	103	1.00 (reference)	0.378
Yes	1	2.45 (0.33 – 18.03)	
MMR status			
pMMR	1	1.00 (reference)	0.690
dMMR	103	20.49 (0.0 – 57948814.58)	
Missing cases	0		
Grade of differentiation			
Well differentiated	6	1.00 (reference)	
Moderately differentiated	57	0.70 (0.20 – 2.43)	0.627
Poorly differentiated	20	0.51 (0.12 – 2.10)	
Missing cases	21		
Mucinous differentiation			
No	13	1.00 (reference)	
Yes, < 50%	18	1.34 (0.37 – 4.86)	0.875
Yes, > 50%	7	1.47 (0.26 – 8.22)	
Missing cases	66		
Lymphangioinvasion			
No	42	1.00 (reference)	0.938
Yes	22	1.04 (0.39 – 2.77)	
Missing cases	40		
Perineural invasion			
No	24	1.00 (reference)	0.613
Yes	7	1.53 (0.29 – 8.04)	
Missing cases	31		

Univariable & multivariable Cox regression analysis included 104 patients.
[a] Analyzed as a continuous variable.
n, number of patients; HR, hazard ratio; 95% CI, 95% confidence interval; NOS, not otherwise specified; pMMR, mismatch repair proficient; dMMR, mismatch repair deficient.

Table S3. Univariable and multivariable Cox proportional hazard models for overall survival for patients with a polyposis syndrome diagnosed with SIA from January 1999 through December 2019 in the Netherlands.

	Univariable			Multivariable		
	n	HR (95% CI)	*p*-value	n	HR (95% CI)	*p*-value
Age, years[a]	41	1.01 (0.98 – 1.05)	0.455			
Sex						
Male	23	1.00 (reference)	0.500			
Female	18	1.33 (0.58 – 3.05)				
Period of diagnosis						
1999-2005	11	1.00 (reference)	0.098			
2006-2012	12	0.93 (0.37 – 2.35)				
2013-2019	18	0.29 (0.09 – 0.97)				
Tumor location						
Duodenum	22	1.00 (reference)	0.732			
Jejunum	11	0.56 (0.20 – 1.53)				
Ileum	4	0.00 (0.00 - NA)				
NOS	4	0.87 (0.25 – 3.06)				
TNM Stage at diagnosis						
Stage I	5	0.05 (0.02 – 0.34)	<0.001	5	0.06 (0.01 – 0.35)	0.012
Stage II	9	0.03 (0.01 – 0.23)		9	0.05 (0.01 – 0.44)	
Stage III	13	0.17 (0.05 – 0.54)		13	0.30 (0.06 – 1.47)	
Stage IV	10	1.00 (reference)		10	1.00 (reference)	
Missing cases	4					
Surgery of the primary tumor						
No	14	1.00 (reference)	<0.001	11	1.00 (reference)	0.243
Yes	27	0.22 (0.09 – 0.53)		26	0.42 (0.10 – 1.80)	
Systemic therapy						
No	33	1.00 (reference)	0.495			
Yes	8	1.49 (0.48 – 4.63)				

Radiotherapy			
No	40	1.00 (reference)	0.158
Yes	1	4.48 (0.56 – 35.83)	
MMR status			
pMMR	8	1.00 (reference)	0.367
dMMR	3	2.47 (0.35 – 17.65)	
Missing cases	30		
Grade of differentiation			
Well differentiated	4	1.00 (reference)	0.003
Moderately differentiated	21	5.31 (0.65 – 43.52)	
Poorly differentiated	6	31.32 (2.99 – 328.64)	
Missing cases	10		
Mucinous differentiation			
No	2	1.00 (reference)	0.720
Yes, < 50%	5	55.94 (0.00 – 15035079483)	
Yes, > 50%	5	1.12 (0.00 - 4848254890.20)	
Missing cases	29		
Lymphangioinvasion			
No	11	1.00 (reference)	0.597
Yes	10	1.55 (0.31 – 7.86)	
Missing cases	20		
Perineural invasion			
No	9	1.00 (reference)	0.779
Yes	4	1.50 (0.09 – 25.39)	
Missing cases	28		

Univariable Cox regression analysis included 41 patients. Multivariable Cox regression analysis included 37 patients, 4 patients excluded due to missings.

[a] Analyzed as a continuous variable.

n, number of patients; HR, hazard ratio; 95% CI, 95% confidence interval; NOS, not otherwise specified; pMMR, mismatch repair proficient; dMMR, mismatch repair deficient.

Table S4. Univariable and multivariable Cox proportional hazard models for overall survival for patients with IBD diagnosed with SIA from January 1999 through December 2019 in the Netherlands.

	Univariable			Multivariable		
	n	HR (95% CI)	*p*-value	n	HR (95% CI)	*p*-value
Age, years[a]	103	1.03 (1.01 – 1.05)	0.006	100	1.03 (1.01 – 1.06)	0.003
Sex						
Male	54	1.00 (reference)	0.806			
Female	49	1.06 (0.66 – 1.72)				
Period of diagnosis						
1999-2005	16	1.00 (reference)				
2006-2012	43	0.59 (0.30 - 1.14)	0.285			
2013-2019	44	0.74 (0.37 – 1.46)				
Tumor location						
Duodenum	16	1.00 (reference)		14	1.00 (reference)	
Jejunum	69	1.02 (0.40 – 2.56)	0.008	7	0.68 (0.23 – 2.05)	0.707
Ileum	7	0.43 (0.23 – 0.81)		68	0.99 (0.47 – 2.06)	
NOS	11	1.04 (0.45 – 2.39)		11	1.38 (0.56 – 3.40)	
TNM Stage at diagnosis						
Stage I	13	0.10 (0.04 – 0.28)		13	0.09 (0.03 – 0.30)	
Stage II	42	0.12 (0.06 – 0.24)	<0.001	42	0.08 (0.03 – 0.22)	<0.001
Stage III	25	0.33 (0.17 – 0.64)		25	0.33 (0.14 – 0.78)	
Stage IV	20	1.00 (reference)		20	1.00 (reference)	
Missing cases	3					
Surgery of the primary tumor						
No	22	1.00 (reference)	0.002	19	1.00 (reference)	0.659
Yes	81	0.42 (0.25 – 0.73)		81	1.20 (0.54 – 2.67)	
Systemic therapy						
No	80	1.00 (reference)	0.495			
Yes	23	1.21 (0.70 – 2.11)				

Radiotherapy			
No	102	1.00 (reference)	0.451
Yes	1	2.15 (0.30 – 15.64)	
MMR status			
pMMR	28	1.00 (reference)	0.528
dMMR	5	0.61 (0.14 – 2.79)	
Missing cases	70		
Grade of differentiation			
Well differentiated	12	1.00 (reference)	0.001
Moderately differentiated	35	2.89 (0.86 – 9.72)	
Poorly differentiated	29	6.55 (1.98 – 22.34)	
Missing cases	27		
Mucinous differentiation			
No	6	1.00 (reference)	0.329
Yes, < 50%	14	2.22 (0.61 – 8.11)	
Yes, > 50%	13	1.22 (0.32 – 4.74)	
Missing cases	70		
Lymphangioinvasion			
No	24	1.00 (reference)	0.027
Yes	29	2.37 (1.10 – 5.09)	
Missing cases	50		
Perineural invasion			
No	10	1.00 (reference)	0.152
Yes	5	3.72 (0.62 – 22.41)	
Missing cases	88		

Univariable Cox regression analysis included 103 patients. Multivariable Cox regression analysis included 100 patients, 3 patients excluded due to missings.

[a] Analyzed as a continuous variable.

n, number of patients; HR, hazard ratio; 95% CI, 95% confidence interval; NOS, not otherwise specified; pMMR, mismatch repair proficient; dMMR, mismatch repair deficient.

Table S5. Univariable and multivariable Cox proportional hazard models for overall survival for patients with celiac disease diagnosed with SIA from January 1999 through December 2019 in the Netherlands.

	Univariable			Multivariable		
	n	HR (95% CI)	*p*-value	n	HR (95% CI)	*p*-value
Age, years[a]	78	1.04 (1.01 – 1.07)	0.017	76	1.04 (1.01 – 1.08)	0.015
Sex						
Male	44	1.00 (reference)	0.554			
Female	34	0.84 (0.47 – 1.50)				
Period of diagnosis						
1999-2005	18	1.00 (reference)	0.914			
2006-2012	26	1.05 (0.52 – 2.12)				
2013-2019	34	0.90 (0.41 – 1.97)				
Tumor location						
Duodenum	30	1.00 (reference)	0.025	28	1.00 (reference)	0.005
Jejunum	34	0.41 (0.22 – 0.79)		34	0.30 (0.14 – 0.63)	
Ileum	7	0.82 (0.28 – 2.38)		7	1.14 (0.36 – 3.59)	
NOS	7	1.29 (0.51 – 3.24)		7	1.51 (0.56 – 4.10)	
TNM Stage at diagnosis						
Stage I	9	0.34 (0.11 – 1.08)	0.041	9	0.72 (0.21 – 2.45)	0.477
Stage II	28	0.33 (0.15 – 0.72)		28	0.81 (0.32 – 2.06)	
Stage III	26	0.48 (0.22 – 1.02)		26	1.35 (0.55 – 3.27)	
Stage IV	13	1.00 (reference)		13	1.00 (reference)	
Missing cases	2					
Surgery of the primary tumor						
No	19	1.00 (reference)	<0.001	18	1.00 (reference)	<0.001
Yes	59	0.25 (0.14 – 0.46)		58	0.26 (0.13 – 0.51)	
Systemic therapy						
No	66	1.00 (reference)	0.152			
Yes	12	0.53 (0.22 – 1.26)				

Radiotherapy			
No	78	NA	NA
Yes	0	NA	
MMR status			
pMMR	8	1.00 (reference)	0.274
dMMR	9	0.45 (0.11- 1.89)	
Missing cases	61		
Grade of differentiation			
Well differentiated	3	1.00 (reference)	
Moderately differentiated	27	1.71 (0.22 – 12.99)	0.854
Poorly differentiated	34	1.78 (0.24 – 13.42)	
Missing cases	14		
Mucinous differentiation			
No	3	1.00 (reference)	
Yes, < 50%	6	1.85 (0.29 – 11.89)	0.190
Yes, > 50%	5	0.29 (0.05 – 1.87)	
Missing cases	64		
Lymphangioinvasion			
No	23	1.00 (reference)	0.396
Yes	15	1.54 (0.57 – 4.15)	
Missing cases	40		
Perineural invasion			
No	8	1.00 (reference)	0.405
Yes	4	2.15 (0.35 – 13.13)	
Missing cases	66		

Univariable Cox regression analysis included 78 patients. Multivariable Cox regression analysis included 76 patients, 2 patients excluded due to missings.
[a] Analyzed as a continuous variable.
n, number of patients; HR, hazard ratio; 95% CI, 95% confidence interval; NOS, not otherwise specified; pMMR, mismatch repair proficient; dMMR, mismatch repair deficient.

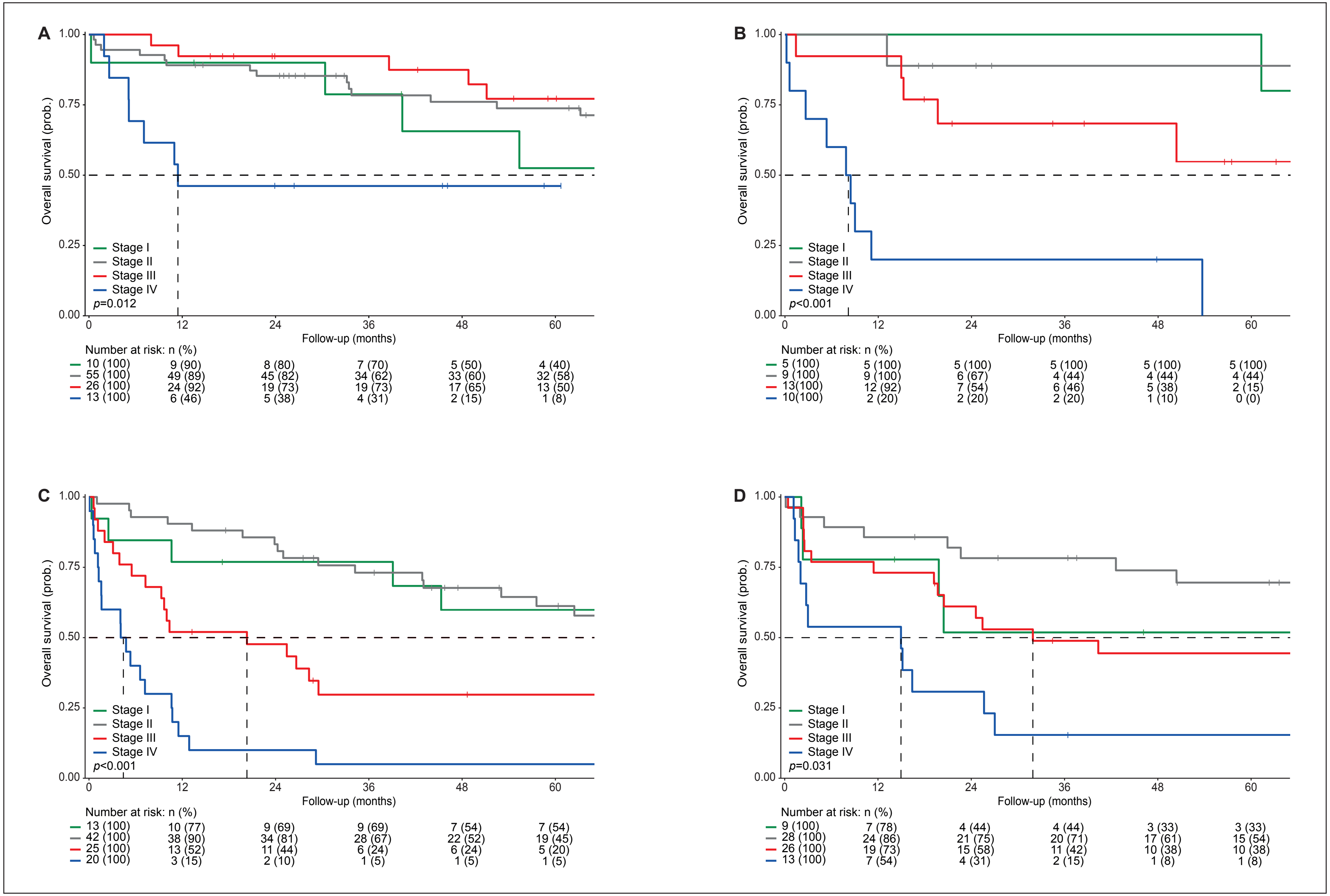

Figure S1. Five-year overall survival per stage of disease of patients diagnosed with SIA and a diagnosis of (A) Lynch syndrome, (B) polyposis syndrome, (C) inflammatory bowel disease and (D) celiac disease. n, number of patients; Prob., probability.
